# Developing a Transnational Health Record Framework with Level-Specific Interoperability Guidelines Based on a Related Literature Review

**DOI:** 10.3390/healthcare9010067

**Published:** 2021-01-13

**Authors:** Ah Ra Lee, Il Kon Kim, Eunjoo Lee

**Affiliations:** 1 School of Computer Science & Engineering, College of IT Engineering, Kyungpook National University, Daegu 41566, Korea; eara0367@gmail.com; 2 College of Nursing, Research Institute of Nursing Science, Kyungpook National University, Daegu 41566, Korea; jewelee@knu.ac.kr

**Keywords:** interoperability, transnational health record, electronic health record, personal health record, health information exchange, health information system

## Abstract

With the advent of digital healthcare without borders, enormous amounts of health information are captured and computerized. As healthcare quality largely depends on the reliability of given health information, personal health records should be accessible according to patients’ mobility, even as they travel or migrate to other countries. However, since all the health information is scattered in multiple places, it is an onerous task to carry it whenever people move to other countries. To effectively and efficiently utilize health information, interoperability, which is the ability of various healthcare information technologies to exchange, to interpret, and to use data, is needed. Hence, building a robust transnational health information infrastructure with clear interoperability guidelines considering heterogeneous aspects is necessary. For this purpose, this study proposes a Transnational Health Record framework, which enables access to personal health records anywhere. We review related literature and define level-specific interoperability guidelines, business processes, and requirements for the Transnational Health Record system framework.

## 1. Introduction

Information and communication technology (ICT) now makes it possible for all health-related information, such as medical records, genomics, lifelogs, and patient-generated data, to be captured and computerized [1]. Diverse stakeholders are interested in using computerized health-related information for personal health, public health, population health, and clinical research [2]. In addition, business enterprises and governments are involved in adopting electronic health record (EHR) systems to improve the quality of healthcare [3]. For example, even patients with the same symptoms can be treated differently depending on their clinical histories. If a physician prescribes medication without knowing a patient’s underlying diseases or drugs being taken, the patient is likely to be exposed to the drug’s side effects [4]. Therefore, the accessibility of reliable health information plays a significant role in healthcare quality, including patients’ safety. For people who travel or migrate to other countries, it would be best for them to take all their health records with them so that they could be prepared for all possible illnesses, accidents, or catastrophic disasters. However, in reality, it is a very onerous task to do so. Hence, building a robust transnational health information infrastructure that can be accessed anywhere would benefit patients and healthcare providers.

For this purpose, healthcare data sharing is a prerequisite [5]. Interoperability issues should be considered first for data sharing because it is difficult for patients to manage their healthcare data only in one place [6]. It is hard to access personal health records outside one’s residence area, so the benefits of computerized health-related information are seriously compromised. More practically, many individuals travel within countries and across borders or migrate to other regions. According to the Medical Tourism Global Market Report by the Market Data Forecast, the global medical tourism market size was about USD 27.8 billion in 2020 and is estimated to be increasing steadily at a compounded annual growth rate (CAGR) of 18.8% to reach USD 65.79 billion by 2025 [7]. However, it is not easy to access personal health records for follow-up treatment after returning to one’s home country due to interoperability issues, such as barriers in language, culture, and medical systems. Thus, interoperability issues should be discussed with careful considerations from a transnational perspective.

Several studies have discussed interoperability issues about healthcare data sharing. However, these studies only focused on technical interoperability, such as data formats, communication channels, and infrastructures. A much more heterogeneous perspective on interoperability is needed to enable seamless transnational healthcare data sharing. In addition, the previous studies also have a limitation in that they were mainly conducted on the integration of various EHR systems within a country [8,9,10,11]. No framework for exchanging health records among different countries exists.

Therefore, this study proposes the novel concept of the Transnational Health Record (THR) system framework, which can access personal health records anytime and anywhere. It is a healthcare information system framework designed to share health information regardless of borders. Considering the fact that interoperability is a prerequisite for data sharing, its importance is much more significant for the THR system, since it should smoothly operate across various countries. Applicable use cases of the THR system would be sharing health information for migrating or traveling to another country, accessing patient summaries in emergencies, transmitting health information for Maritime Telemedical Assistance Services, managing personal health records in My Health Data Bank, or monitoring health information in a Digital Companion. A Digital Companion is a personal secretary that helps people acquire information via digital devices and gives them psychological comfort. These benefits cannot be achieved without sharing health information across countries with different languages, cultures, policies, and regulations.

This study defines a framework for the THR system with level-specific interoperability guidelines. We analyze related literature to find what interoperability factors need to be considered for the level-specific guidelines. In addition, we derive business processes and requirements for the THR system framework.

### Interoperability Levels

Interoperability is the ability of healthcare information technology to exchange, to interpret, and to use data seamlessly. Digitalization makes it easy for all health information to be captured and computerized. The lack of interoperability is a major obstacle to health-related data sharing with different institutions, regions, or even countries. Interoperability includes comprehensive capabilities, such as a data format, structure, syntax, communication protocol, business process, and policy. It is a critical factor for moving ahead to the digital healthcare environment.

Several studies analyzed interoperability in multilayered aspects [12,13,14,15,16,17]. These are in various domains, such as in military, industry, IT systems, crisis management, and public administration. Each study presents interoperability levels according to their domain-specific environments. Even in different domain environments, there is something in common. Most studies have the technical interoperability level because it is the basic requirement for transmitting data electronically among different information systems. The higher levels deal with elements that require more effort to satisfy according to domain-specific characteristics. For example, organizational interoperability includes legal, political, or even cultural aspects of the institutions that participate in data sharing. Since the level-specific requirements are different according to the domain characteristics, the lack of clear guidelines for a particular domain makes barriers to the interoperability of information systems.

Lehne et al. studied digital medicine’s interoperability specifically in the healthcare domain [17]. This study emphasizes the importance of interoperability in digital medicine and provides four aspects of interoperability: technical, syntactic, semantic, and organization. Technical interoperability ensures information exchange requirements between different systems. With advances in ICT, achieving technical interoperability, including securely transferring and receiving data with communication protocols, is usually straightforward. Syntactic interoperability is about data syntax, and semantic interoperability concerns medical terminology. International standards development organizations, such as Health Level Seven (HL7), Integrating the Healthcare Enterprise (IHE), and the World Health Organization (WHO), support syntactic and semantic interoperability by specifying data formats, structures, syntax, models, and codification. For example, Fast Healthcare Interoperability Resources (FHIR) could enhance syntactic interoperability, and Logical Observation Identifier Names and Codes (LOINC) could be used for semantic interoperability. Table 1 shows the healthcare standards classified by the Healthcare Information and Management Systems Society (HIMSS) [18].

## 2. Materials and Methods

To propose the novel concept of the THR system, this study consists of three parts. First, we review related studies. We search and analyze the related literature on exchanging health information between different institutions in the same country or across borders in Europe. This part shows which studies are close to our THR concept and what they identify as challenging interoperability issues. Second, based on the results of the literature analysis, we define the level-specific interoperability guidelines necessary for the THR system. In addition, we derive requirements and business processes by addressing open issues for the THR system.

### Strategies to Search for Related Studies

**Figure 1:**
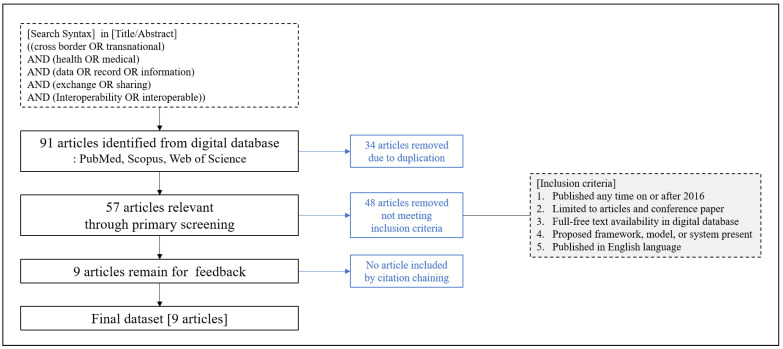
Selection criteria for previous studies on health information exchange systems.

The objective of our THR system is to enable access to personal health records anywhere. To exchange health records with different countries, various aspects of interoperability should be taken into consideration. However, to the best of our knowledge, no study exists that takes analytical considerations or systematical frameworks for the THR system into account. To derive suitable elements for the THR system, we review the literature on the exchange of health information across borders or regions. Figure 1 shows the selection criteria for reviewing previous studies. We selected five main bundles of keywords to find related studies. The first group of words comprises “cross-border” or “transnational”. The second bundle includes “health” or “medical”. The third consists of “data”, “record”, or “information”. The words in the fourth group are “exchange” or “sharing”. Finally, “interoperability” or “interoperable” are in the fifth bundle. We searched three databases (Web of Science, PubMed, and Scopus) to see if titles and abstracts contained the selected keywords. At first, 91 articles were selected by electronically searching for combinations of the keywords, but 34 articles were removed due to duplication. Then, we reviewed each article’s abstract manually to exclude those whose study purpose was not closely related to ours. The inclusion criteria for the selection of previous studies were: (1) published any time in or after 2016, (2) limited to articles and conference papers, (3) full free text available in a digital database, (4) present a framework, a model, or a system architecture, and (5) written in English. Finally, nine articles were selected to review the interoperability issues [19,20,21,22,23,24,25,26,27].

## 3. Results

### 3.1. Analysis of the Selected Literature Using Interoperability Levels

We analyzed the nine selected works from the literature with the four interoperability levels [17]. Table 2 shows that all the selected articles discussed technical interoperability. Syntactic and semantic interoperability was also mostly considered. However, only three articles addressed organizational interoperability issues, such as cultural, political, and legal factors, but not entirely.

Organizational interoperability is the main barrier to enhancing healthcare services’ quality. Especially in healthcare, it is not well defined and is poorly understood by all stakeholders, such as healthcare providers, patients, IT professionals, caregivers, and government officials. Organizational interoperability has many factors to consider. It is also difficult to clearly understand its concepts because it is associated with a wide range of areas, including language, culture, laws, and policies. Despite the need for a profound analysis of organizational interoperability, it has not yet been sufficiently researched in the healthcare domain.

All three papers defined requirements for organizational interoperability. However, these are focused on a European exchange, cloud, and security. They did not consider business processes and workflows. Business process interoperability is crucial, since it is a building block of the THR system’s service requirements. It also addresses organizational interoperability concerns. Each country has different healthcare systems, languages, cultures, policies, and regulations. Without consideration of business processes for organizational interoperability, the THR system could not effectively allow local medical staff to respond when an emergency occurs in a place that is outside of a patient’s original residential area. For seamless exchange of health information, it needs to take the business process interoperability into account so that medical staff can quickly handle patients from different backgrounds and environments.

### 3.2. Level-Specific Interoperability Guidelines

**Figure 2:**
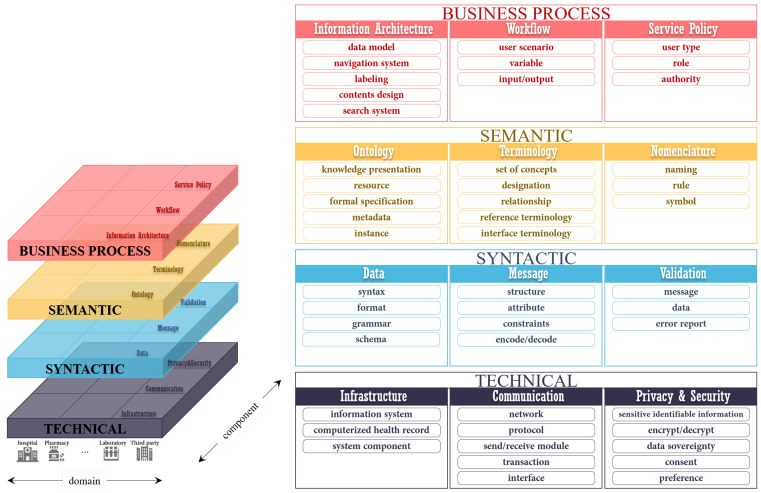
Layered level-specific interoperability.

We propose level-specific interoperability guidelines for the THR system. These consist of four levels: technical, syntactic, semantic, and business processes. The considerations of each level were derived from the literature analysis results. The remarkable point of the level-specific interoperability guidelines proposed in this study is that the guidelines deal with business process interoperability. This is associated with the functional aspects, such as workflow, that must be defined to share healthcare data between different countries effectively. The business process interoperability level contributes to solving the current challenging issue—the lack of organizational interoperability. Figure 2 shows the level-specific interoperability layers.

#### 3.2.1. Technical Interoperability

Technical interoperability is the ability to transmit and receive data between different information systems. It is the most well studied of the interoperability levels. The technology for exchanging data electronically in various domains, such as e-government and military, has already been sufficiently applied. Technical interoperability is not difficult to achieve with enough previous studies. Health information must be computerized for a seamless exchange of health records. In addition, IT infrastructure, such as a healthcare information system, is necessary. Those involved are encouraged to improve existing health IT infrastructures—thus increasing interoperability step by step—to the extent possible. Building a new IT infrastructure or developing a new health information system is expensive. Communication networks, channels, interfaces, and protocols for interactions are also required for technical interoperability. Selecting an appropriate communication environment for handling health data is vital because health records include sensitive personal information. Two major aspects of technical interoperability in the healthcare domain are privacy and security. The public’s trust that personal health records in the THR system are safely secured must be obtained. Many countries are striving to place appropriate, healthy, and adequate safeguards for personal health records as interoperability increases across nations in order to acquire such trust. Personal health records should support transparency regarding the business processes that access their data, with consideration of patients’ preferences.

#### 3.2.2. Syntactic Interoperability

Syntactic interoperability is the definition of a specific format of the information to be exchanged. The structures and attributes should be standardized to satisfy syntactic interoperability. It is also necessary to define a format for actual data exchange, such as XML. Reusability is a significant element that researchers should consider in syntactic aspects. It should not simply end at electronically exchanging data between different information systems with syntactic interoperability for interpretation. In digital healthcare circumstances, health information could bring significant results when transferred to other systems and reused. The international standard that has outstanding reusability is the HL7 FHIR. It is considered as the next-generation healthcare information standard framework. Clinical Document Architecture (CDA), the previous development standard of HL7, operates on a document basis. On the other hand, FHIR uses a concept called a “resource” to express a small unit of data needed in the healthcare environment. Resources increase the reusability of electronically recorded health information. For example, suppose that a Care Record Summary is registered to a health information system in the CDA. In that case, the data must be manually extracted and rewritten if only drug prescriptions are to be used for other purposes. On the other hand, in FHIR, each piece composing the Care Record Summary is divided into resources so that the necessary parts can be extracted and reused. In addition, the syntactic interoperability should include a data validation process related to the format, syntax, grammar, or schema.

#### 3.2.3. Semantic Interoperability

Compared to technical or syntactic interoperability, semantic interoperability is more domain specific and depends on the service contents. In the healthcare domain, various terminologies are used for each field, such as diagnosis, examination, dentistry, and nursing. These terms should be able to be interpreted and understood with the same meaning anywhere. Semantic interoperability is about making sure that the shared information has the same meanings between different institutions or countries. There are different vocabularies of specific codes for clinical concepts. Any person reading a medical document could get the meaning that the writer intended, even though the document was not written in style or with words that the reader is not accustomed to. Health information must be universally understood. As different countries may have differences in even the same medical documents, it is necessary to define a standardized format that can be unified internationally. International standards provide such vocabularies. The LOINC code is a representative example that can be used to ensure semantic interoperability. In addition, language compatibility is required. Health information can be written in different languages, such as Japanese or Arabic, using different alphabet characteristics. All the participating countries or institutions need to use a common glossary to interpret the shared information. In the semantic area, well-defined metadata could be a critical factor for ensuring semantic interoperability successfully. The metadata enable effective management of personal health records, such as registration, identification, and searching.

#### 3.2.4. Business Process Interoperability

Business process interoperability supports the functions required by the THR system. It focuses on the mechanical processing of the service for each workflow. A service-oriented perspective of inter-organizational processes could be useful for achieving business process interoperability. This level is something that has not been considered enough compared to the other three interoperability levels. Health information exchange involving multiple countries brings users together from various environments. Accessing health records in the THR system should be done in the same manner in all environments, even with patients from different countries. Business process interoperability requires the definition of the information architecture and workflow necessary for system services, such as user management, data registration, and access. Like the semantic level, it is domain specific. To ensure business process interoperability, different countries should come to an agreement regarding the THR system, and global cooperation within the public sector is required. The business process interoperability could prevent errors in preferential mistakes due to cultural differences between countries. It is a straightforward subject of discussion when making policy agreements or regulations regarding the THR system.

We derive several requirements for the THR system from the main actions. First, it is necessary to identify and manage users, as with all information systems. Since the THR system aims to gain accessibility to health information anytime and anywhere, users should be managed at a transnational level. The THR-ID, an international patient identifier, is required to identify and manage registered users globally. The THR-ID could be issued when a user first enrolls in the THR system. It must be a unique value, such as a combination of a passport number, country code, and the issue date. Like general portal sites, an ID and password are also needed for login. In addition, it is necessary to register personal health records in the THR system. For example, a patient could ask a physician to register their allergy information in the THR system. Another main action is accessing personal health records in the THR system. In an emergency, while on a business trip to another country, a local paramedic could access a patient’s allergy information through the THR system. There are other open issues relevant to applying the THR systems in real-life environments. How can patients who got caught in an accident be identified in an emergency when they have communication problems? For instance, what could the patients do if they have difficulty breathing in a country where the people use a different language? What type of data should be shared through the THR system? Who is involved in the personal health information sharing process? How is the audit process for sharing data containing sensitive personal information performed? How is the consent to provide personal health records given?

However, the THR system can be used not only for emergencies, but also for other purposes. First, in terms of self-healthcare, a person may get medical treatment or discover an illness abroad during a business trip. These histories would be recorded in the THR system, and even when the patient returns to their home country, it should be possible to continue healthcare by using these records. The second is the use of research data. In the case of rare and incurable diseases, medical researchers usually do not have a sufficient amount of data. However, with the THR system, the researchers would have access to more case data from various countries that could be shared and used for research purposes. It should be possible to authorize data providers, that is, patients, to use their data in specific institutions. Table 3 and Table 4 show the lists of business processes and the THR system requirements.

### 3.3. Framework for the THR System

**Figure 3:**
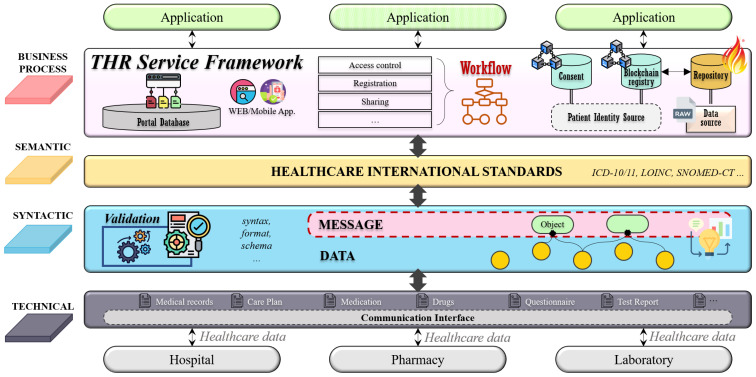
Layered system architecture for level-specific interoperability in the THR framework.

This study proposes a framework for the THR system following the level-specific interoperability guidelines defined above. Figure 3 shows an overview of the THR system framework. This THR framework consists of layers that support the four levels of interoperability defined in Figure 2. The technical layer supports communication interfaces that can be shared between other institutions or countries. Through communication interfaces, it is possible to transmit and receive healthcare data between different institutions. The syntactic layer is related to the interoperability regarding the data syntax, format, and schema. This layer validates whether a message created through the combination of raw data satisfies the syntactic feature. The semantic layer ensures interoperability for ontological parts by utilizing international healthcare standards. Finally, the business process layer supports business processes and workflows of THR services. This layer contains access control, registration, and services, which are useful functions for THR users.

**Figure 4:**
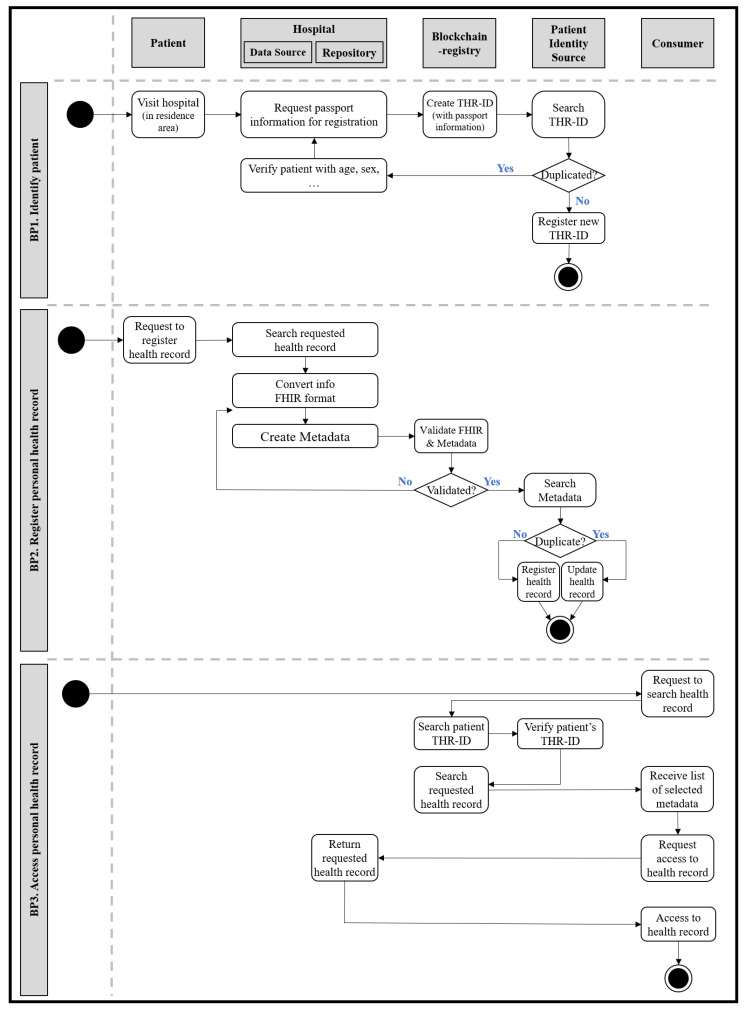
Workflows for business processes in the THR framework.

The THR framework’s components consist of five system actors: data source, repository, consumer, patient identity source, and blockchain registry. These five components are based on IHE Cross-Enterprise Document Sharing (IHE XDS.b) [28], and each system actor’s role and exchange interactions are defined as standard. Figure 4 shows an example of workflows for the THR framework. It represents the business processes defined above.

The patient identity source manages identifiers of system-wide users: patients. It is associated with adding new patients or searching for existing users. The data source refers to an information system storing raw data—computerized personal health records. In the data source, data will be formalized and standardized. In order to satisfy syntactic and semantic interoperability, various international healthcare standard technologies and terminologies are applied. For example, FHIR could be used as the primary data model, and the LOINC code could express the data attribute values. In addition to the standardization of the data, the creation of metadata is also performed in the data source. Metadata contain a brief overview of standardized data. They are created and delivered to the repository along with standardized data. The repository is in charge of permanently storing original standardized data. Only data that comply with the specified data format and standard can be stored. The data that do not conform to the format cannot pass validation and are not saved. If the data format validation is successful, the original data will be stored in the repository, and the metadata are sent to the blockchain registry. The registry is a repository that manages the metadata to be shared, and only data stored there can be exchanged. The consumer consumes data and searches for the desired data in the registry. For example, if the consumer wants to have a specific patient’s health record for the past month, the consumer would search the registry for the patient’s identifier and set the searching period as parameters. The registry returns a list of metadata that meet the conditions set by the consumer, and these metadata contain a reference address to the repository where the original standardized data are stored. Consumers can obtain the desired data through the repository reference address in the finally returned metadata.

This framework has a little differentiation from XDS, which is the application of blockchain technology. A blockchain registry is formed to construct a blockchain network between institutions participating in the exchange of health records. Information stored in the blockchain cannot be changed or deleted, and all transactions occurring in the network are recorded transparently. There are two advantages to this. First, it ensures data integrity. In the metadata, there is a field that stores the hash value of the original standardized data. Since this value is stored in the blockchain, it is impossible to manipulate the data once they are stored. After the consumer accesses the repository and receives the desired data, the consumer can easily check whether the data are genuine by comparing the hash value of the data with the hash field value in the metadata. Second, all transactions related to metadata are transparently recorded in the blockchain registry. Since all blockchain network activities are monitored, accountability traceability can be secured. So, any access with malicious intent can be prevented.

## 4. Discussion

This study proposes the novel concepts of a THR system framework and level-specific interoperability guidelines for the THR system. The THR system proposed in this study has three main contributions. The first is the level-specific interoperability guidelines. The lack of interoperability is a significant issue in digital healthcare. The core of digital healthcare is data. These big healthcare data are scattered across various hospitals, pharmacies, institutions, and even countries. Careful consideration of interoperability is a key factor in exchanging data among diverse institutions that have different data formats, structures, languages, or even cultural aspects. The need for interoperability has already been recognized for a long time, and many related studies have also been conducted [5]. However, previous studies have mostly focused on technical interoperability, such as with data formats [19,20,21,22,23,24,25,26,27]. Interoperability includes not only the technical aspects, but also business processes, workflows, and policies. It should be discussed in multilayered factors. This study sets four interoperability levels to ensure the completeness of interoperability in the healthcare data sharing process. In addition, it provides guidelines for each level-specific requirement. The level-specific interoperability guidelines contribute to a heterogeneous approach to interoperability problems, which are currently limited in technical aspects.

The second is a THR system that enables access to personal health records anytime and anywhere, regardless of borders. With the advent of a digital society, various information is being shared around the world. Likewise, data sharing among different healthcare service institutions is essential for efficiently and effectively using personal healthcare data that are scattered in multiple places. However, previous studies are limited to data sharing with different institutions or regions in the same country. They usually focus on technical, syntactic, or semantic integration of different EHR systems. Interoperability issues, however, should be dealt with more comprehensively by taking organization interoperability into account. With organizational interoperability, personal health records among different countries could be integrated more easily with the THR system. The THR system provides various benefits, including boosting global medical tourism, improving how medical staff deal with emergencies, and preparing for catastrophic disasters. It also can create an international medical research environment by sharing cases of rare and intractable diseases worldwide. Ultimately, the THR system improves public health, reduces medical costs, and is useful in international cooperative research environments.

Finally, in this study, business processes and requirements for the THR system are derived. The THR system is a novel concept allowing people around to have access to their health information anytime and anywhere. It involves exchange of data between countries with different languages, cultures, and policies. Therefore, it is necessary to consider a straightforward business process that can overcome possible problems caused by diversity in patients’ living environments.

In the European e-government framework, the need for organizational interoperability has already been recognized and considered [29]. Kubicek, H. et al. [30] analyzed the definition of organizational interoperability in various e-government frameworks and concepts. They divided organizational interoperability into three views: functional, institutional, and IT service. The scope of organizational interoperability is broad, and includes social, cultural, and legal factors. Thus, organizational interoperability is not simply an upper layer of the technical, syntactic, and semantic operability, but is interrelated with all interoperability levels. The business process interoperability alone could be the upper layer in the functional view. From each of the three viewpoints, ICT has sufficient capacity to exchange data between different information systems at a transnational level. In addition, international healthcare standards, such as FHIR and SNOMED-CT, are actively being developed to express healthcare terminologies in unified formats. The convergence of ICT and healthcare standards could make it relatively easy to ensure interoperability in the IT service view.

In the institutional view, organizational interoperability depends on the executives at all levels who are willing to cooperate with other nations for shared gains. Political aspects could be resolved through an agreement between nations participating in the exchange of health information. To come to an agreement transnationally, it is imperative to clearly define subjects to be discussed. The business process interoperability, including workflows, patient identification, registration, authentication, data access control, consent, and history management, should be defined first. Business processes make participating organizations coordinate workflows, data models, management, and system services. As many interactions would occur, designing and defining business processes give more benefits to THR system users by consolidating all the participants’ value arrangements in a collaboration network. Business process interoperability is a basis for an agreement on shared goals and ground rules for achieving mutual benefits. This study derives business processes, requirements, and workflows for the THR system.

Our study has limitations, since it focuses on the business process interoperability, which is a part of organizational interoperability. Political aspects, such as regulations and legal issues, are not discussed in this study. Nevertheless, we believe that the business process should be considered first to reach a political consensus between countries, as it could identify and address necessary factors of the THR system.

As a part of future work, a minimal data-set should be discussed for utilizing health information through the THR system. The THR system aims to provide a high quality of healthcare services to patients worldwide by sharing healthcare data across countries. The minimal data-set should be uniform and contain the minimum health records needed by any healthcare service provider. Since there are discrepancies in each country’s healthcare systems and processes, it is essential to define a standard data model or a minimal data-set. A summary history of a patient’s health should be interoperable so that healthcare providers in different environments know the person’s health status in order to avoid jeopardizing the patient’s safety. The HL7 International Patient Summary (IPS) can be used as a minimal data-set for the THR system. It is a minimal and non-exhaustive patient summary that is readily usable by all clinicians for unscheduled care, including for patients from different countries [31]. The IPS emphasizes the need to provide generic solutions for global application beyond a particular region or country. As the data-set of the IPS is intended for global use, it is suitable for the THR system. The IPS profile contains a full mapping of the ISO/EN 17269 data elements for international healthcare standards—CDA and FHIR. The IPS consists of four sections, which include required fields, such as medication history or allergies. The IPS is sustainable in ensuring clinical validity by meeting clinical workflow, documentation, and information queries. In the case of FHIR, 18 resources (Patient, Observation, Practitioner, MedicationStatement, Medication, AllergyIntolerance, Condition, Immunization, Procedure, Organization, DeviceUseStatement, Device, Specimen, Imaging Study, DiagnosticReport, CarePlan, Consent, and Composition) are used to implement the contents of the IPS.

In addition, in further studies, we plan to conduct a user experience evaluation for users who may use this system. The THR system has a variety of stakeholders, including patients, medical staff, and researchers. Further research should discuss such user experience opinions from various stakeholders and should continuously search for further improvements.

## 5. Conclusions

In this study, we proposed a novel concept of a THR system framework based on level-specific interoperability guidelines, including the business process, for organizational interoperability. To the best of our knowledge, there has been no research on a THR system that exchanges health information globally. This study contributes to a new THR concept framework for making shareable healthcare information accessible anytime and anywhere. To effectively and efficiently utilize health records regardless of borders, it is necessary to consider broad aspects of interoperability at the technical, syntactic, semantic, and organizational levels. Primarily, we expect that our results will provide important insight into organizational interoperability. Definite business processes and workflows, new level-specific interoperability guidelines, and a methodological framework for the THR system resulted from this study. It ensures interoperability among various countries with different cultures and healthcare systems.

## Figures and Tables

**Table 1:** Healthcare standards.

Category	Standard
Vocabulary and Terminology	Current Procedural Terminology (CPT) Healthcare Common Procedure Coding System The International statistical classification of diseases and related health problems (ICD-10/11) Logical Observation Identifier Names and Codes (LOINC) National Drug Code (NDC) National Library of Medicine drug name/vocabulary normalization (RxNorm) Systematized Nomenclature of Medicine–Clinical Terms (SNOMED-CT)
Content	HL7 Version 2.X (V2) HL7 Version 3 Clinical Document Architecture (CDA) Consolidated CDA (C-CDA) Fast Healthcare Interoperability Resources (FHIR)
Transport	Digital Imaging and Communications in Medicine (DICOM) IHE specifications (e.g., XDS, XDR, XDM)
Privacy and Security	Health Insurance Portability and Accountability Act (HIPAA) General Data Protection Regulation (GDPR)
Identifier	Enterprise Master Patient Index (EMPI) Medical Record Number (MRN) National Council of State Boards of Nursing ID (NCSBN ID) National Provider ID (NPI) Object ID (OID)

**Table 2:** Results of the analysis of the related literature using interoperability levels.

Previous Study	Technical	Syntactic	Semantic	Organizational
Neville, K. et al. (2016) [19]	X	X	X	
Staffa, M. et al. (2018) [20]	X	X		
Kinsner-Ovaskainen, A. et al. (2018) [21]	X	X	X	
Natsiavas, P. et al. (2018) [22]	X	X		X
Nalin, M. et al. (2019) [23]	X	X	X	X
von Martial, S. (2019) [24]	X	X	X	
Gavrilov, G. et al. (2019) [25]	X	X		
Otero Varela, L. et al. (2020) [26]	X	X	X	
Aarestrup, F. et al. (2020) [27]	X			X

**Table 3:** Business process for the Transnational Health Record (THR) system.

ID	Business Process	Description
BP1	Identify patient	Patient is identified and authenticated via a transnationally issued ID
BP2	Register personal health record	Practitioner registers health record
BP3	Access personal health record	Practitioner retrieves health record
BP4	Manage personal health record	Patient manages their own health record status
BP5	Grant access to health record	Patient grants institutions access to personal health record

**Table 4:** Requirements for the THR system.

ID	Requirements	Description
RQ1	User credentials	THR-ID and password for login, electronic card for authentication
RQ2	Minimal data-set	Common required data for health information, such as a patient summary
RQ3	Personal health record	Main asset to be exchanged through the THR system

## Data Availability

Not applicable.

## References

[1] Raghupathi W., Raghupathi V. (2014). Big Data Analytics in Healthcare: Promise and Potential. Health Inf. Sci. Syst..

[2] Kukafka R. (2019). Digital Health Consumers on the Road to the Future. J. Med. Internet Res..

[3] Shin S. (2019). Current Status and Future Direction of Digital Health in Korea. Korean J. Physiol. Pharmacol..

[4] Aust S., Schwameis R., Gagic T., Müllauer L., Langthaler E., Prager G., Grech C., Reinthaller A., Krainer M., Pils D. (2020). Precision Medicine Tumor Boards: Clinical Applicability of Personalized Treatment Concepts in Ovarian Cancer. Cancers.

[5] Hulsen T. (2020). Sharing Is Caring—Data Sharing Initiatives in Healthcare. Int. J. Environ. Res. Public Health.

[6] Kolasa K., Kozinski G. (2020). How to Value Digital Health Interventions? A Systematic Literature Review. Int. J. Environ. Res. Public Health.

[7] Global Medical Tourism Market Size, Share, Trends, Growth & COVID-19 Analysis Report. https://www.marketdataforecast.com/market-reports/medical-tourism-market.

[8] Dinh-Le C., Chuang R., Chokshi S., Mann D. (2019). Wearable Health Technology and Electronic Health Record Integration: Scoping Review and Future Directions. JMIR mHealth uHealth.

[9] Campbell A., McCarty D., Rieckmann T., McNeely J., Rotrosen J., Wu L., Bart G. (2019). Interpretation and Integration of the Federal Substance Use Privacy Protection Rule in Integrated Health Systems: A Qualitative Analysis. J. Subst. Abus. Treat..

[10] Garcia S., Wortman K., Cella D., Wagner L., Bass M., Kircher S., Pearman T., Penedo F. (2019). Implementing Electronic Health Record–Integrated Screening of Patient-Reported Symptoms and Supportive Care Needs in a Comprehensive Cancer Center. Cancer.

[11] Evans R. (2016). Electronic Health Records: Then, Now, and in the Future. Yearb. Med. Inform..

[12] Tolk A., Muguira J.A. The levels of conceptual interoperability model. Proceedings of the 2003 Fall Simulation Interoperability Workshop.

[13] Gottschalk P. (2009). Maturity Levels for Interoperability In Digital Government. Gov. Inf. Q..

[14] Guédria W., Naudet Y., Chen D. (2013). Maturity Model for Enterprise Interoperability. Enterp. Inf. Syst..

[15] Rezaei R., Chiew T., Lee S. (2014). An Interoperability Model for Ultra Large Scale Systems. Adv. Eng. Softw..

[16] Campos C., Chalmeta R., Grangel R., Poler R. (2013). Maturity Model for Interoperability Potential Measurement. Inf. Syst. Manag..

[17] Lehne M., Sass J., Essenwanger A., Schepers J., Thun S. (2019). Why Digital Medicine Depends on Interoperability. NPJ Digit. Med..

[18] Interoperability in Healthcare. https://www.himss.org/resources/interoperability-healthcare.

[19] Neville K., O’Riordan S., Pope A., Rauner M., Maria R., Madden M., Sweeney J., Nussbaumer A., McCarthy N., O‘Brien C. (2016). Towards the Development of a Decision Support System for Multi-Agency Decision-Making during Cross-Border Emergencies. J. Decis. Syst..

[20] Staffa M., Sgaglione L., Mazzeo G., Coppolino L., D’Antonio S., Romano L., Gelenbe E., Stan O., Carpov S., Grivas E. (2018). An Openncp-Based Solution for Secure Ehealth Data Exchange. J. Netw. Comput. Appl..

[21] Kinsner-Ovaskainen A., Lanzoni M., Bubenheim M., Martin S. (2018). A Sustainable Solution for the Activities of the European Network For Surveillance of Congenital Anomalies: EUROCAT as Part of the EU Platform on Rare Diseases Registration. Eur. J. Public Health.

[22] Natsiavas P., Rasmussen J., Voss-Knude M., Votis K., Coppolino L., Campegiani P., Cano I., Marí D., Faiella G., Clemente F. (2018). Comprehensive User Requirements Engineering Methodology for Secure and Interoperable Health Data Exchange. BMC Med. Inform. Decis. Mak..

[23] Nalin M., Baroni I., Faiella G., Romano M., Matrisciano F., Gelenbe E., Martinez D., Dumortier J., Natsiavas P., Votis K. (2019). The European Cross-Border Health Data Exchange Roadmap: Case Study in the Italian Setting. J. Biomed. Inform..

[24] Von Martial S., Brix T., Klotz L., Neuhaus P., Berger K., Warnke C., Meuth S., Wiendl H., Dugas M. (2019). EMR-Integrated Minimal Core Dataset for Routine Health Care and Multiple Research Settings: A Case Study for Neuroinflammatory Demyelinating Diseases. PLoS ONE.

[25] Gavrilov G., Vlahu-Gjorgievska E., Trajkovik V. (2019). Healthcare Data Warehouse System Supporting Cross-Border Interoperability. Health Inform. J..

[26] Otero Varela L., Le Pogam M., Metcalfe A., Kristensen P., Hider P., Patel A., Kim H., Carlini E., Perego R., Gini R. (2020). Empowering Knowledge Generation through International Data Network: The IMeCCHI-DATANETWORK. Int. J. Popul. Data Sci..

[27] Aarestrup F., Albeyatti A., Armitage W., Auffray C., Augello L., Balling R., Benhabiles N., Bertolini G., Bjaalie J., Black M. (2020). Towards a European Health Research and Innovation Cloud (HRIC). Genome Med..

[28] IHE Cross-Enterprise Document Sharing. https://wiki.ihe.net/index.php/Cross-Enterprise_Document_Sharing.

[29] Kubicek H., Cimander R. (2009). Three dimensions of organizational interoperability. Eur. J. ePract..

[30] Kubicek H., Cimander R., Scholl H. (2011). Organizational Interoperability in E-Government.

[31] HL7 IPS International-Patient-Summary.net. http://international-patient-summary.net.

